# How workplace loneliness harms employee well-being: A moderated mediational model

**DOI:** 10.3389/fpsyg.2022.1086346

**Published:** 2023-01-16

**Authors:** Ameer A. Basit, Shazia Nauman

**Affiliations:** ^1^GIFT Business School, GIFT University, Gujranwala, Pakistan; ^2^Riphah School of Business and Management, Riphah International University (Lahore), Lahore, Punjab, Pakistan

**Keywords:** workplace loneliness, job satisfaction, need to belong, work engagement, well-being, moderated-mediation

## Abstract

This study investigated the effect of workplace loneliness on work-related subjective well-being by proposing work engagement as an explanatory mechanism in the workplace loneliness—job dissatisfaction relationship. Moreover, the study examines the need to belong as a coping mechanism in the relationship between workplace loneliness and work engagement. Specifically, the study posits that workplace loneliness reduces the positive and fulfilling state of work engagement that in turn increases job dissatisfaction and that this mediation depends on the employee’s level of need to belong. Data were collected from employees (*N* = 274) working in diverse domestic and multinational organizations in Lahore, Pakistan. Results showed that workplace loneliness reduced the work engagement of lonely individuals that in turn increased their job dissatisfaction. However, the deleterious effect of workplace loneliness on work engagement was weaker for individuals having a higher need to belong. These findings have important implications for organizations wishing to mitigate the harmful effects of workplace loneliness on employees’ subjective well-being.

## Introduction

In inclusive organizations, people are valued regardless of their group membership, status, or individual differences. Inclusive workplaces align and make use of employees’ talents through their broad participation and the systems driven by equity. However, this desired state of the inclusive organization is seriously hampered when there is a deficiency of strong and stable relationships among organizational members making them feel lonely and excluded. Workplace loneliness is dissatisfaction in social relationships at the workplace (Lam and Lau, 2012) and is important for employees’ personal and workplace life (Wright, 2012). Employees spend substantial time at the workplace, however, it is not well understood how deprivation of their belongingness needs affect various workplace outcomes and what coping mechanisms can help to mitigate its negative outcomes (Anand and Mishra, 2021). Although the detrimental effects of loneliness on people have been extensively studied in psychology, research on employee workplace loneliness is at an early stages in the organizational behavior literature (Ozcelik and Barsade, 2018) and requires further investigation.

Psychologists believe that, like physical pain, loneliness is a social pain causing serious harm to the cognition, emotion, behavior, and well-being of individuals (Kalil et al., 2010). Research focusing on the outcomes of workplace loneliness has revealed its deleterious effects on various employee outcomes. For instance, where lonely employees demonstrate a decrease in creativity (Peng et al., 2017), in-role and extra-role performance (Lam and Lau, 2012; Ozcelik and Barsade, 2018), affective organizational commitment (Ozcelik and Barsade, 2018), they also show increase in job burnout (Omar et al., 2020; Anand and Mishra, 2021), intention to leave (Chen et al., 2016), and unethical behavior (Gentina et al., 2018). The Covid−19 pandemic has forced many employees performing jobs in different professions to work from home and remain in social isolation thus deteriorating their well-being (Gabr et al., 2021; Nishimura et al., 2021; Chirico et al., 2022). Thus, further investigation is needed as how workplace loneliness negatively affects employees’ work-related well-being, which is an underexplored area in organizational research (Anand and Mishra, 2021; Andel et al., 2021; Wright and Silard, 2021). To address this gap, we examined how workplace loneliness enhances job dissatisfaction which is detrimental for both the employees and the organizations and results in turn over and work withdrawal (Zhou and George, 2001).

Drawing from the employee engagement theories (Kahn, 1990; Bakker and Demerouti, 2008), this study aims to investigate whether workplace loneliness impacts the work-related well-being of employees by decreasing their work engagement and consequently increasing their job dissatisfaction. Researchers are interested to identify factors that help to tone down the negative effects of workplace loneliness (Andel et al., 2021). In response to this call, we introduce need to belong as a moderator in the workplace loneliness—work engagement relationship and contend that the need to belong is highly relevant personal resource that helps employees to cope with work loneliness. Employing social reconnection theory (Maner et al., 2007), we investigate whether the need to belong, as a personal resource and an individual difference, interacts with workplace loneliness to influence work engagement. This study deepens our understanding of how and when the need to belong would weaken the negative relationship between workplace loneliness and work engagement.

**Figure 1:**
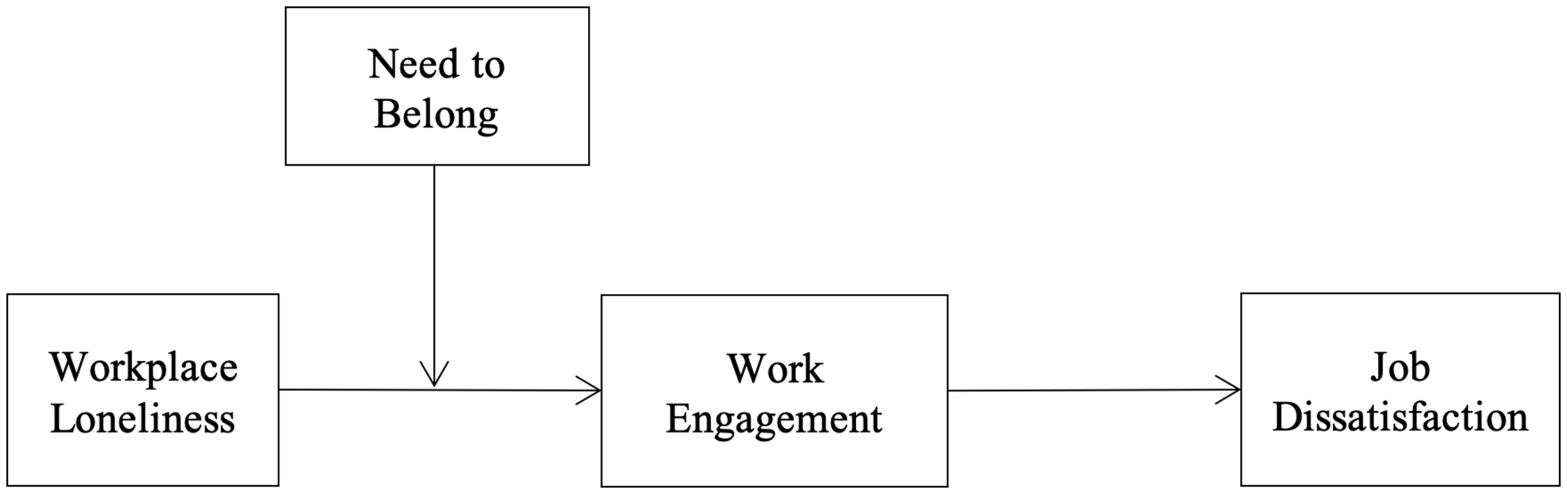
Conceptual model.

We aim to broaden the understanding of workplace loneliness by making mainly three contributions to the HR literature. First, by examining the mediational role of work engagement in the loneliness—dissatisfaction relationship (Anand and Mishra, 2021), we advance organizational research by explaining why lonely individuals are dissatisfied with their work. Second, we respond to the calls by earlier researchers to investigate factors that can help employees to tone down the effects of workplace loneliness (Andel et al., 2021; Wright and Silard, 2021). Third, this study was conducted in a developing country that has a collectivist culture and is an underrepresented region in management research. To address these questions, this study thus proposes and tests a moderated mediational model of workplace loneliness that is rooted in the engagement and social reconnection theories (Figure 1).

## Theory and hypotheses

### Workplace loneliness

Human beings have a natural need for social interaction and close relationships with others. However, individuals experience loneliness if these needs are not sufficiently met (Baumeister and Leary, 1995; Wright and Silard, 2021). Workplace loneliness is defined as “employees’ subjective affective evaluations of, and feelings about, whether their affiliation needs are being met by the people they work with and the organizations they work for” (Ozcelik and Barsade, 2018). It is often the quality rather than the frequency of interpersonal relationships that leads to feelings of loneliness (Wright et al., 2006). In another study, Azambuja and Islam (2019), found that middle managers often experience alienation due to competing work demands and diverse roles, leading to a lack of personal connections and belonging within their organization. It is important to note that although it may appear similar, workplace loneliness is distinct from concepts such as isolation, aloneness, and solitude as it is a subjective psychological state rather than an objective characteristic of an individual’s social environment (Wright et al., 2006; Ozcelik and Barsade, 2018).

The above evidence thus clearly shows that workplace loneliness is a disturbing social aspect of the workplace that needs more attention in research to understand how inclusive organizations can be developed. This study focuses on how loneliness affects the well-being of individuals at work, such as work engagement and job dissatisfaction, and how the need to belong can influence the deleterious effects of workplace loneliness on well-being.

### Workplace loneliness and work engagement

According to McKee (2017), the quality of social interaction with colleagues is crucial for bolstering employee happiness and work engagement because employees feel frustrated when they find themselves lonely, invisible to others, and unnoticed. Work engagement refers to the positive and fulfilling work-related mental state characterized by vigor, dedication, and absorption in work (Schaufeli et al., 2002). Employee engagement researchers consider the quality of interpersonal relationships at the workplace an important source of work engagement (Kahn, 1990; Halbesleben, 2010; Christian et al., 2011). For instance, Kahn (1990), argues that interpersonal relationships with superiors and co-workers and leadership style promote work engagement by enhancing psychological safety, which is the perception of the consequences of taking interpersonal risks at the workplace (Edmondson and Lei, 2014). Psychological safety is associated with work engagement in empirical research (May et al., 2004; Halbesleben, 2010).

It can be argued that workplace loneliness is likely to decrease work engagement by reducing psychological safety because lonely employees have a fear of rejection by others and consider it risky for their status in the organization by putting in any further efforts to build relationships with others (Kahn, 1990). Evidence shows that quality relationships reflected by organizational, supervisory, and coworker support are related to work engagement *via* psychological safety (May et al., 2004; Rich et al., 2010). It is thus likely that the deficiency of high-quality relationships as reflected by the subjective experience of loneliness will reduce work engagement through a decline in psychological safety.

Another stream of engagement research rooted in the job demands-resources theory (Hakanen et al., 2006; Bakker and Demerouti, 2008; Bakker and Bal, 2010; Hu et al., 2011) argue that healthy interpersonal relationships with supervisors and colleagues are job resources that promote work engagement by helping employees to achieve their work goals, to reduce job demands, and to stimulate their personal growth, learning, and development. For instance, the relationship between social support from supervisors and work engagement has been found in several empirical studies with a strong association of social support with dedication followed by the vigor and absorption dimensions of work engagement (Schaufeli and Bakker, 2004; Hakanen et al., 2006; Llorens et al., 2006; Bakker et al., 2007; Xanthopoulou et al., 2007; De Lange et al., 2008; Schaufeli et al., 2008; Hu et al., 2011). It can thus be expected that as a deficient job resource, workplace loneliness depletes the cognitive and emotional energies of employees needed to achieve work goals and personal development and makes them withdraw from their work engagement consequently. In addition, there is evidence that the experience of loneliness shatters one’s self-confidence and fosters stress and fear (Hornstein and Eisenberger, 2022). Low self-confidence makes individuals doubt their abilities to perform at work and stress mars their engagement. According to Kahn (1990), low confidence reduces one’s psychological availability (i.e., feeling able to use physical, emotional, and cognitive resources at work) that in turn decreases work engagement.

In addition, evidence shows that a work environment that is deficient in opportunities to integrate and relate with others results in distress and makes individuals withdraw and disengage themselves from the such environment to alleviate their loneliness (Russell et al., 1984; Rastogi et al., 2018). Based on the aforementioned logic and empirical evidence, we propose that:

*Hypothesis 1*: Workplace loneliness is negatively related to work engagement.

### Work engagement and job dissatisfaction

Work engagement and job satisfaction are distinct job attitudes (Christian et al., 2011; Albrecht et al., 2015). Therefore, in their causal model of work engagement, Albrecht et al. (2015) and Gong et al. (2020) have mentioned job satisfaction as a positive individual outcome of work engagement. When employees can develop energetic and affective connections with their work life, they engage and deal well with their job demands (Schaufeli et al., 2008). According to Saks (2006), work engagement is associated with employee attitude, behaviors, and intentions at work. Studies have reported that engaged employees are more satisfied, motivated, and committed to their jobs with lower intentions to quit as compared to disengaged employees (Demerouti et al., 2001; Schaufeli and Bakker, 2004). The cost of bearing a disengaged employee has made understanding the consequences of work engagement even more important (Fleming et al., 2005; Rayton et al., 2012).

Engaged employees expect positive work outcomes and can satisfy their needs by engaging themselves in their work (Mauno et al., 2007). Their high level of engagement enables them to experience positive emotions (Schaufeli and Van Rhenen, 2006) and develop positive feedback that fulfills their needs for appreciation, recognition, and success (Bakker and Demerouti, 2008). However, when employees fail to engage with their work then their work life becomes a repetition of motion and certain practices (Hollis, 2015). According to Kahn (1990), actively disengaged employees seem to be physically present yet psychologically absent from their work, they remain unhappy and share unhappiness with others. Such individuals dwell in a withdrawal state where their creativity declines (De Castella and Byrne, 2015), and job dissatisfaction amplifies (Carnahan, 2013). It is thus proposed that:

*Hypothesis 2*: Work engagement is negatively related to job dissatisfaction.

### The mediating role of work engagement

According to Baumeister and Leary (1995), the unmet social needs of individuals threaten their sense of personal significance and deteriorate their well-being. Such employees are reluctant to emotionally invest their selves in their work (Ozcelik and Barsade, 2011), making them ultimately dissatisfied with their job. We expect that work engagement will mediate the relationship between workplace loneliness and job dissatisfaction because employees want to develop an emotional attachment to and engage their selves with their work through feelings of warmth, belongingness (Meyer et al., 1997), and relatedness (Ryan and Deci, 2000), and they report negative attitudes when such positive experiences do not occur at the workplace (Saks, 2006).

The mediating role of work engagement in the relationship between workplace loneliness and job dissatisfaction is more evident when we view it from the perspective of Kahn’s (1990) theory of employee engagement who argued that the social relations at the workplace determine how safe (or unsafe) employees psychologically feel while engaging in their work roles. Thus, as a result of loneliness, employees’ affiliation needs remain unmet and they are likely to feel psychologically unsafe to express and employ their authentic selves fully at work, leading to their reduced engagement in work roles (Kahn, 1990; May et al., 2004; Rich et al., 2010). Based on the social and emotional needs approach, the typology of loneliness by Weiss (1987), explains that when individuals value a network of relationships where they can integrate and share an interest with others and when they feel valued in their work environment, they tend to fully engage themselves at their workplace (Crawford et al., 2010). Prior researchers (Saks, 2006; Moura et al., 2014; Gong et al., 2020), found that work engagement was related to job satisfaction because when employees experienced their work engagement as fulfilling and positive, they were more likely to report positive attitudes toward their jobs and organizations. Because loneliness erodes the positive and fulfilling experience of work engagement, employees are likely to report dissatisfaction with their jobs. We thus propose that:

*Hypothesis 3*: Work engagement mediates the positive relationship between workplace loneliness and job dissatisfaction.

### The moderating role of need to belong

We have argued above that workplace loneliness reduces work engagement that in turn causes job dissatisfaction among lonely employees. This argument implies that these harmful effects will similarly exist for all lonely individuals. However, we believe that not all individuals are equally harmed by their loneliness because people differ in responding to their social pain of loneliness as a function of their unique individual differences, such as the need to belong. Researchers view the need to belong as a fundamental human motivation (Baumeister and Leary, 1995) and failure to sufficiently fulfill this need amplifies feelings of isolation and loneliness (Mellor et al., 2008). However, we believe that people differ in the degree of this need and the effect of loneliness on their work engagement will depend on whether they have a high or low need to belong.

Earlier studies have linked the need to belong and loneliness with social and psychological well-being (Zumaeta, 2019; Oyanedel and Paez, 2021). Satisfying the need for belongingness is viewed as an antecedent to healthy social ties and a buffer against perceived isolation (Mellor et al., 2008). Lonely employees assume that their colleagues and organization are unable to fulfill their affective needs which makes them reluctant to invest their selves emotionally at work (Ozcelik and Barsade, 2011). Such perceptions influence one’s performance, as individuals with satisfied affective needs work harder (Hackett et al., 1994), while loneliness triggers work withdrawal (Ozcelik and Barsade, 2011; Yilmaz, 2011).

In their social reconnection theory, Maner et al. (2007) note that social exclusion stimulates a desire to reconnect with others because of the need to belong which is a fundamental human motivation behind forming interpersonal relationships. These authors acknowledged and found support that the social exclusion–outcome relation is constrained by various boundary conditions, such as fear of negative evaluation and anticipated interaction with the new partner. Building on Maner et al. (2007), social reconnection argument in the context of work engagement, we contend that not all individuals experience decline in their work engagement due to having a deficiency in their social relationships. The detrimental effect of loneliness on work engagement is less salient for individuals who are high on their need to belong because they exercise alternative strategies to stimulate their pro-social behaviors to build and revive relationships with colleagues and their need to belong pushes them to stay engaged as a means of inclusion in the social interaction process through high performance (Baumeister and Leary, 1995; Maner et al., 2007; Gentina et al., 2018). In contrast, individuals with less need to belong are more likely to detach from their work and will have lesser work engagement. Based on this rationale, we propose that:

*Hypothesis 4*: Need to belong moderates the negative relationship between workplace loneliness and work engagement such that the relationship is weaker when the need to belong is higher than when it is lower.

## Method

### Sample and procedure

This study was carried out on a wide and diverse range of public, private, and not-for-profit organizations located in Lahore, Pakistan. Most of these companies had offices in a large government-owned software technology park. Among 90 companies that were contacted for this study, 78 agreed to participate. We sent e-mail invitations containing a link to our online survey to the contact persons of these companies who then forwarded the links to their employees. In total, 600 invitations were sent and 290 responses were received, yielding a response rate of 48%. After the initial screening, 274 responses were finalized for the main analysis. Table 1 shows that a majority of the respondents were male (75%), single (67%), had earned master’s degrees (56%) and worked in managerial positions (64%). Most of our respondents were employed in private sector organizations (58%) and an average respondent was 28.5 years (SD = 6.1) old.

**Table 1:** Sample demography.

	*N*	%
Gender		
Male	206	75.2
Female	68	24.8
Marital status		
Single	184	67.2
Married	90	32.8
Education		
Master	154	56.2
Bachelor	109	39.8
Diploma	9	3.3
Doctorate	2	0.7
Position		
Non-managerial	99	36.1
First-line management	88	32.1
Middle management	76	27.7
Top management	11	4.00
Sector		
Private	160	58.4
Public	92	33.6
Not-for-profit	22	8.0
	Mean (SD)	Range
Age	28.5 (6.1)	19.0–67.0

### Measures

Using self-reported items, respondents indicated the extent of their agreement with each item on a five-point Likert scale ranging from *strongly disagree* (1) to *strongly agree* (5) for the scales of workplace loneliness, job dissatisfaction, and need to belong. However, for assessing work engagement, a six-point scale was used that ranged from *almost never* (1) to *always* (6).

**Workplace loneliness.** A 20-item UCLA Loneliness Scale developed by Russell et al. (1980), was used. A sample item is “I lack companionship at my work.” The Alpha reliability of this scale was 0.90.

**Job dissatisfaction.** We measured job dissatisfaction with a three-item scale from the Michigan Organizational Assessment Questionnaire (Seashore et al., 1982). Following (Zhou and George, 2001), we reverse-coded the items such that higher scores indicated greater job dissatisfaction. A sample item is “In general, I do not like my job.” The Alpha reliability of the scale was 0.77.

**Need to belong.** We used a 10-item scale of Leary et al. (2013), to measure the need to belong. Three reverse-scored items showed poor reliability and therefore were removed from the analysis. A sample item is “I want other people to accept me.” The Alpha reliability of the scale was 0.76.

**Work engagement.** We assessed work engagement using a three-item ultra-short version of the Utrecht Work Engagement Scale (UWES-3) developed by Schaufeli et al. (2019). These items are “At my work, I feel bursting with energy,” “I am enthusiastic about my job,” and “I am immersed in my work.” The Alpha reliability of this scale was 0.71.

## Results

### Descriptive statistics and correlations

Table 2 presents the descriptive statistics and correlations among study variables. Workplace loneliness significantly correlated to work engagement (*r* = −0.37, *p* < 0.001) and job dissatisfaction (*r* = 0.62, *p* < 0.001). Work engagement significantly correlated to job dissatisfaction (*r* = −0.43, *p* < 0.001). Need to belong, however, showed a non-significant correlation with workplace loneliness, work engagement, and job dissatisfaction. Among the demographic variables, only education and position showed significant correlations with some of our study variables, therefore, their effects were controlled during the main analyses. Furthermore, results of the independent sample t-test showed that the perceptions of male and female participants about loneliness, belongingness, engagement, and dissatisfaction were not statistically different from each other.

**Table 2:** Descriptive statistics, zero-order correlations, and reliabilities.

Variable	Mean	SD	1	2	3	4	5	6	7	8
1. Job dissatisfaction	2.07	0.87	(0.77)							
2. Workplace loneliness	2.34	0.65	0.62^***^	(0.90)						
3. Need to belong	3.34	0.72	−0.02	0.06	(0.76)					
4. Work engagement	4.25	1.07	−0.43^***^	−0.37^***^	0.04	(0.71)				
5. Vigor	3.93	1.42	−0.18^**^	−0.24^***^	−0.01	0.77^***^				
6. Dedication	4.56	1.31	−0.47^***^	−0.34^***^	0.10	0.82^***^	0.43^***^			
7. Absorption	4.27	1.29	−0.38^***^	−0.30^***^	0.01	0.79^***^	0.37^***^	0.55^***^		
8. Education	3.53	0.61	−0.05	0.02	0.11	0.13^*^	0.07	0.11	0.13^*^	
9. Position	3.00	0.90	0.05	−0.04	−0.14^*^	−0.19^**^	−0.12^*^	−0.14^*^	−0.18^**^	−0.26^***^

*N* = 274. Values along the diagonal are Alpha reliabilities.

*p < 0.05;

**p < 0.01;

***p < 0.001.

### Common method bias test

We performed Harman’s single-factor test Podsakoff et al. (2003), to assess the presence of common method bias. All items of the four constructs were forced to load on a single un-rotated factor that extracted only 26% of the variance, indicating that much of the variance was not captured by this single factor. This showed that the common method bias did not influence our findings.

### Confirmatory factor analysis

To reduce the complexity of the measurement model, we maintained a favorable parameter-to-sample size ratio and developed a just-identified measurement model using the item parceling method as suggested by methodologists (Landis et al., 2000; Bandalos, 2002; Little et al., 2013). We parceled items of workplace loneliness and need-to-belong scales into three by averaging the items having the highest and lowest factor loadings and repeated this process until we created three item parcels for both these constructs. The CFA results showed that the measurement model achieved a good model fit (*χ*^2^(48) = 94.8, *p* < 0.001; RMSEA = 0.06; SRMR = 0.05; TLI = 0.95; CFI = 0.97). Further, factor loadings of all indicators on their respective constructs were above the conservative cutoff value of 0.50 (workplace loneliness = 0.84–0.90; need to belong = 0.66–0.73; work engagement = 0.52–0.82; job dissatisfaction = 0.60–0.84). Furthermore, the alpha reliabilities (0.71–0.90) and construct reliabilities (0.72–0.90) were above 0.70. These results provided evidence that the measurement model achieved the convergent validity.

Table 3 presents the discriminant validity evidence. The proposed four-factor measurement model showed best model fit [*χ*^2^(48) = 94.8, *p* < 0.001; RMSEA = 0.06; SRMR = 0.05; TLI = 0.95; CFI = 0.97] as compared to all other nested models where relationships were constrained. As compared to all other models, the single factor model (*χ*^2^(58) = 856.3, *p* < 0.001; RMSEA = 0.23; SRMR = 0.33; TLI = 0.32; CFI = 0.40) showed the poorest model fit. These results demonstrated that the hypothesized four-factor model achieved discriminant validity.

**Table 3:** Discriminant validity with comparison of alternative measurement models.

Model	*χ* ^2^	*df*	Δ*χ*^2^	Δ*df*	RMSEA	SRMR	TLI	CFI
Four-factor (hypothesized)	94.76	48	–	–	0.06	0.05	0.95	0.97
Three-factor (combined NTB and WE)	348.83	51	254.07^***^	3	0.15	0.16	0.71	0.78
Three-factor (combined WL and NTB)	444.65	51	349.89^***^	3	0.17	0.18	0.62	0.70
Three-factor (combined WL and WE)	460.48	51	365.72^***^	3	0.17	0.25	0.60	0.69
Two-factor (combined WL, NTB, and WE)	751.11	54	656.35^***^	6	0.22	0.31	0.36	0.47
One-factor (combined all)	856.34	58	761.58^***^	10	0.23	0.33	0.32	0.40

NTB, Need to belong; WE, Work engagement; WL, Workplace loneliness.

***p < 0.001.

### Results of hypotheses testing

We tested our moderated mediation model using Model 7 of the Process procedure for SPSS (Hayes, 2013) controlling for education and position. Results showed that workplace loneliness had a significant negative effect on work engagement (*b* = −0.63, *p* < 0.001), lending support to Hypothesis 1. Work engagement had a significant negative effect on job dissatisfaction (*b* = −0.18, *p* < 0.001), providing support to Hypothesis 2. The direct effect of workplace loneliness on job dissatisfaction was significant (*b* = 0.72, *p* < 0.001), showing the possibility of the partial mediating effect of work engagement in the relationship between workplace loneliness and job dissatisfaction. We used the bias-corrected bootstrapping method by generating 5,000 bootstrap samples for a 95% level of confidence for the confidence intervals to test the significance of the proposed mediation. Results showed that the mediation effect of work engagement was significant (effect size = 0.11, 95% CI [0.05, 0.18]) because the confidence intervals did not include the value of zero (Hayes, 2013). Thus, Hypothesis 3 was supported.

**Figure 2:**
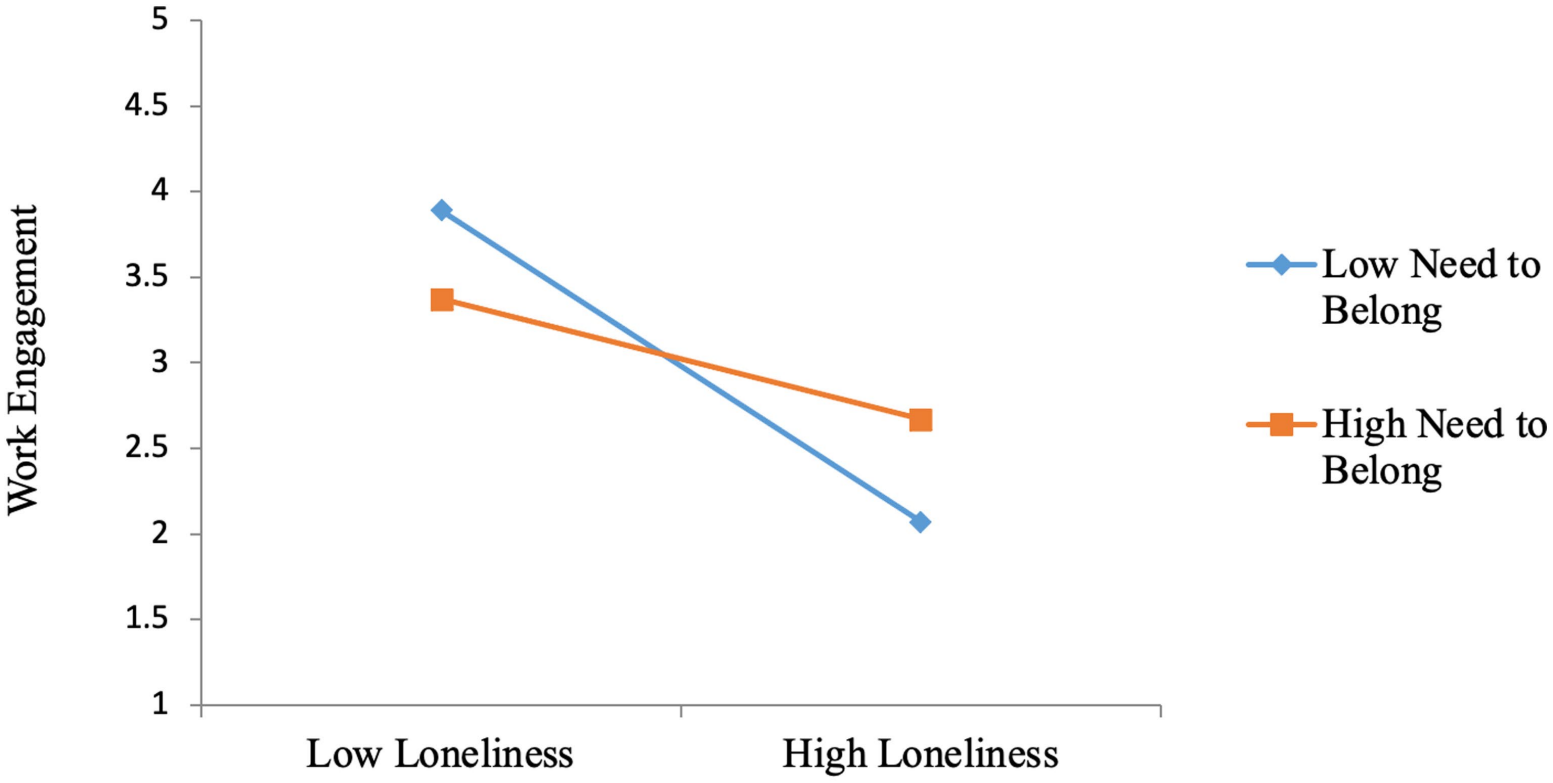
Combined effects of workplace lonelines and need to belong on work engagement.

Next, the results showed a significant combined effect of workplace loneliness and the need to belong on work engagement (*b* = 0.28, *p* < 0.01). A slope test was performed to verify the nature of this interaction. As shown in Figure 2, individuals who had a higher need to belong continued to demonstrate high work engagement despite their loneliness. This showed that a higher need to belong weakened the deleterious effect of workplace loneliness on work engagement among these individuals. Thus, Hypothesis 4 was supported.

Finally, we tested the conditional indirect effect of workplace loneliness on job dissatisfaction *via* work engagement while taking into account the moderating effect of the need to belong. Table 4 shows that the effect of workplace loneliness on job dissatisfaction *via* work engagement was weaker (effect size = 0.08, 95% CI [0.03, 0.14]) when the need to belong was higher than when it was lower (effect size = 0.15, 95% CI [0.07, 0.24]). The index of moderated mediation (effect size = −0.05, 95% CI [−0.10, −0.01]) revealed that the conditional indirect effect of workplace loneliness on job dissatisfaction was statistically significant. Thus, the proposed moderated mediation model received significant statistical support.

**Table 4:** Regression results for conditional indirect effects.

	Work engagement		Job dissatisfaction	
	*b*	SE	*B*	SE
Education	0.19	0.11	−0.02	0.07
Position	−0.19^**^	0.07	0.03	0.05
Workplace loneliness	−0.63^***^	0.09	0.72^***^	0.07
Need to belong	0.02	0.08		
Workplace loneliness × need to belong	0.28^**^	0.11		
Work engagement			−0.18^***^	0.04
*R^2^*	0.20		0.43	
∆*R^2^*	0.02^**^		0.23^***^	
Conditional indirect effects	Indirect Effect/Index	SE	95% CI (Lower, Upper)	
Need to belong				
−1 SD (−0.72)	0.15	0.05	0.07, 0.24	
Mean (0)	0.11	0.03	0.05, 0.18	
+1 SD (0.72)	0.08	0.03	0.03, 0.14	
Index of moderated mediation	−0.05	0.02	−0.10, −0.01	

*N* = 274. Unstandardized regression coefficients are reported. Bootstrap sample size = 5,000. CI, confidence interval.

**p < 0.01;

***p < 0.001.

## Discussion

This study aimed to address the effect of workplace loneliness on job dissatisfaction *via* work engagement along with the moderating role of the need to belong. We tested our moderated mediation model among 274 Pakistani employees who worked in diverse organizations. The findings of this study confirm that workplace loneliness increases job dissatisfaction among lonely employees by decreasing their engagement with work and that this indirect effect of workplace loneliness is weaker when lonely employees have a higher need to belong.

Our findings are in line with previous research showing that a discrepancy between the desired and existing levels of interpersonal relationships makes employees feel lonely and distanced at their workplaces (Wright et al., 2006; Hawkley et al., 2010; Ozcelik and Barsade, 2018). Employees’ desire to have fulfilling relationships at work makes them feel valued, significant, and satisfied with their jobs (Tabancali, 2016). However, loneliness snaps their self-confidence and cultivates stress (Hawkley et al., 2010), generating feelings of doubt and frustration. Because of being unable to satisfy their social need for belongingness at work, lonely employees become demotivated, disengaged, and dissatisfied with their work (Bakker and Demerouti, 2008).

Work engagement is associated with desirable employee attitudes and behaviors (Saks, 2006), as they can satisfy their needs through effective engagement at work (Mauno et al., 2007). Our results support the evidence that for disengaged employees their work routine becomes a mere repetition of work-related behaviors that are devoid of social or emotional connectivity toward work (Hollis et al., 2015). Such employees remain physically present yet psychologically absent and live in a withdrawal state of dissatisfaction toward their work (Carnahan, 2013).

The findings of the present study also revealed that work engagement mediates the relationship between workplace loneliness and job dissatisfaction. These findings indicate that loneliness alienates and isolates employees from their environment, making them personally frustrated and less approachable toward colleagues. Such a work climate is deficient in opportunities for employees to connect and integrate at the workplace, reducing their engagement and commitment toward work (Schaufeli and Bakker, 2004; Bakker and Bal, 2010). Employees who are unable to interpersonally integrate at work perceive their selves unnoticed and invisible to others, leading them to demonstrate poor performance, low productivity, and job dissatisfaction (Akçit and Barutçu, 2017).

Furthermore, this study has found that the unmet need for belongingness poses a threat to one’s well-being, whereas the satisfied need for belongingness acts as a buffer against the deleterious effects of workplace loneliness (Mellor et al., 2008). Our findings suggest that employees with a higher need to belong are less affected by their loneliness as compared to the employees with a lower need to belong because they do not try to reconnect with others and rather prefer to detach from their group and work as a means of reaction to their loneliness. Our findings are consistent with the previous research, suggesting that individuals with satisfied and high affective needs work harder (Hackett et al., 1994; Mellor et al., 2008), while loneliness triggers withdrawal and dissatisfaction (Tabancali, 2016; Wright and Silard, 2021). In sum, this study was directed to study relatively less explored phenomena of workplace loneliness and its effects on employees’ work-related well-being.

### Contribution to theory and practice

This study contributes to knowledge in several ways. It enhances our understanding of employee loneliness in the domain of work in particular, and reveals it as a barrier for inclusive organizations in general. The role of social needs in the workplace is commonly ignored despite having clear evidence pointing toward its impact on employees’ work outcomes (Zhou and Wu, 2018). The findings of our study augment the existing workplace loneliness literature by exploring the previously unexplored moderating role of the need to belong and mediating role of work engagement in the organizational context. We respond to the calls for research (Anand and Mishra, 2021; Andel et al., 2021; Wright and Silard, 2021), by examining work engagement as a mediator and the need to belong as a moderator in the work loneliness–job dissatisfaction relationship, thus extending the nomological network of workplace loneliness. Furthermore, this study provides evidence for the prevalence of workplace loneliness in the collectivist culture of Pakistan where social bonds are expected to be stronger as compared to the individualistic cultures. This implies that loneliness is a universal social issue having serious consequences for the well-being of its victims.

This study has practical implications for managers to consider. Rapid industrialization and globalization have made organizational cultures overwhelmingly competitive and demanding. The competitive work environments can generate performance in the short term but ignoring workplace loneliness can result in reduced employee well-being and ultimately poor job performance in the long run. Thus, managers seeking to make their organizations effective and inclusive should try to enhance friendly social interactions among colleagues over and above their individual differences. They should align and make use of the talents of their diverse workforce through their broader participation and the systems are driven by equity. Further, to tone down the negative effects of workplace loneliness, HR may consider the need to belong as an important factor in the selection process specifically for desk jobs that require less social interaction. HR should create socialization activities for such employees and provide social support to make them feel socially connected. Moreover, organizations need to pay special attention to employees having a low need to belong and be responsive to workplace loneliness by being more inclusive in their relationship with their employees. Further, both HR and organizations may focus on improving the quality of working conditions which may help to reduce employees’ loneliness.

### Strengths, limitations, and future directions

This study has both strengths and limitations. The occupational diversity of our sample enhances and extends the generalizability of our findings to the broader population of Pakistani employees. In addition, this study produces evidence from a country of 200 million, which has a collectivist culture and is an underrepresented region in management research. A major limitation of this study is its cross-sectional design which decreases our absolute confidence in the causal direction of our constructs although our logical argument supports the causal direction. We suggest that future research should employ time-lagged, longitudinal, or field experiment designs to check if they predict different findings. Furthermore, as loneliness indicates the misfit between one’s existing and desired social needs, it would be interesting to view this concept through the lens of the person-environment fit theory (Kristof-Brown et al., 2005; Follmer et al., 2018), to explore more mediating and moderating conditions of the workplace loneliness–outcomes relations.

## Conclusion

Modern technology has made possible instant connections and interpersonal communication between hundreds of employees, yet workplace loneliness is on the rise (Mell et al., 2018). Contemporary organizations are seeking to become inclusive where individuals can comfortably use their full potential toward achieving goals and can enhance their well-being. However, workplace loneliness impedes this path and calls for sound management interventions to make organizations more inclusive.

## Data availability statement

The raw data supporting the conclusions of this article will be made available by the authors, without undue reservation.

## Ethics statement

The studies involving human participants were reviewed and approved by The Research Ethics Committee of GIFT University. The patients/participants provided their written informed consent to participate in this study.

## Author contributions

AB: designed the study, reviewed literature, collected data, and prepared the first draft. SN: reviewed literature and actively participated in writing and finalizing the manuscript. All authors contributed to the article and approved the submitted version.

## Conflict of interest

The authors declare that the research was conducted in the absence of any commercial or financial relationships that could be construed as a potential conflict of interest.

## Publisher’s note

All claims expressed in this article are solely those of the authors and do not necessarily represent those of their affiliated organizations, or those of the publisher, the editors and the reviewers. Any product that may be evaluated in this article, or claim that may be made by its manufacturer, is not guaranteed or endorsed by the publisher.
